# Development of Torque Sensor with High Sensitivity for Joint of Robot Manipulator Using 4-Bar Linkage Shape

**DOI:** 10.3390/s16070991

**Published:** 2016-07-01

**Authors:** Hong-Xia Zhang, Young-Jae Ryoo, Kyung-Seok Byun

**Affiliations:** 1 Department of Mechanical Engineering, Mokpo National University, Jeonnam 585543, Korea; addy-zhang@hotmail.com; 2 Department of Control Engineering and Robotics, Mokpo National University, Jeonnam 58554, Korea; yjryoo@mokpo.ac.kr

**Keywords:** torque sensor, robot manipulator, joint torque control, 4-bar linkage

## Abstract

The torque sensor is used to measure the joint torque of a robot manipulator. Previous research showed that the sensitivity and the stiffness of torque sensors have trade-off characteristics. Stiffness has to be sacrificed to increase the sensitivity of the sensor. In this research, a new torque sensor with high sensitivity (TSHS) is proposed in order to resolve this problem. The key idea of the TSHS comes from its 4-bar linkage shape in which the angular displacement of a short link is larger than that of a long link. The sensitivity of the torque sensor with a 4-bar link shape is improved without decreasing stiffness. Optimization techniques are applied to maximize the sensitivity of the sensor. An actual TSHS is constructed to verify the validity of the proposed mechanism. Experimental results show that the sensitivity of TSHS can be increased 3.5 times without sacrificing stiffness.

## 1. Introduction

Industrial robot manipulators have been widely used to improve productivity in various industries such as automotive, electronics and shipbuilding. To increase the intelligence of the robot manipulator, many kinds of sensors have been developed. Among these, sensors to measure force and torque have been increasingly used in robot manipulators. These sensors can improve the performance of the robot manipulator [1].

Forces and torques of end effector and joint torques are related by the Jacobian matrix as follows [2]

(1) $$\begin{array}{c} \Gamma =J^{T}F\\ F={\left[F_{x},F_{y},F_{z},T_{x},T_{y},T_{z}\right]}^{T}\\ \Gamma ={\left[T_{1},T_{2},T_{3},T_{4},T_{5},T_{6}\right]}^{T} \end{array}$$

where, *T_1_ −T_6_* are the torque at the joints of the manipulator. *F_z_, F_y_, F_z_, T_x_, T_y_, T_z_* are forces and torques at the end effector of the manipulator. *J* is the Jacobian matrix of the manipulator.

**Figure 1:**
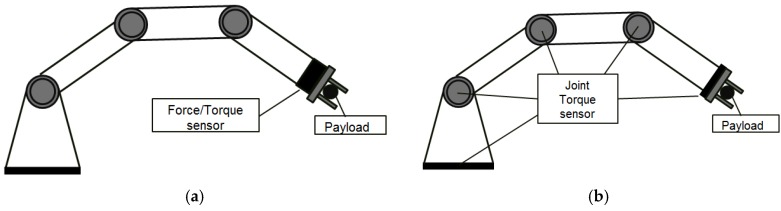
Force and torque sensors for manipulator. (**a**) 6-axis FT(force/torque) sensors; (**b**) joint torque sensors.

**Figure 2:**
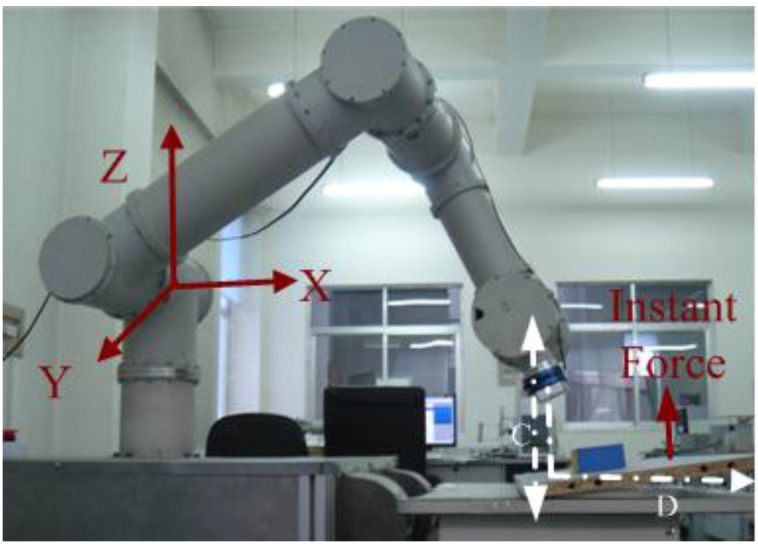
Manipulator working with FT sensor [3].

As shown in Figure 1, the sensors to measure force and torque can be classified as 6-axis FT (force/torque) sensors and joint torque sensors. The 6-axis FT sensors (Figure 1a) are attached to the end-effector. It can directly and precisely measure the reaction forces and torques between the manipulator and the environment which the manipulator contacts with. The 6-axis FT sensors are small and sensitive. For example, as shown in Figure 2, the FT sensor JR3 [3] is used at the end effector of the robot manipulator. However, this sensor only can measure the force/torque at the distal end of the manipulator. It cannot detect the conflict or external forces acting on the manipulator body. As a result, an FT sensor attached at the end effector of a manipulator cannot be used to improve the safety of working environments when humans and robots are working in the same space.

On the other hand, joint torque sensors (Figure 1b) measure torque forces at joints. The joints of a robot manipulator generally consist of an actuator, a reducer, an encoder, a break, a bearing, etc. Joint torque sensor can be added such as a DLR (Deutsches Zentrum für Luft- und Raumfahrt e.V.; German Aerospace Center), joint of manipulators LWRIII [4]. Feedback from the joint torque sensors can be used to compensate for the nonlinearities and modeling uncertainties of manipulator dynamics [5]. Furthermore, joint torque sensors can prevent accidents or reduce damage by controlling the manipulator force. This can lead to the improved intelligence of robot manipulators.

Nowadays, more and more robots are working together with people. These kinds of robots should have safety functions to prevent collision accidents in an unstructured real environment [6]. Safety-based design of the manipulator can involve a FT sensor which measure forces and torques at the end-effector of the robots [7]. However, when a collision is caused by other parts of the arm, human safety cannot be ensured [8]. Therefore in this study, the joint torque sensor used for joints of the robot manipulator is discussed.

**Figure 3:**
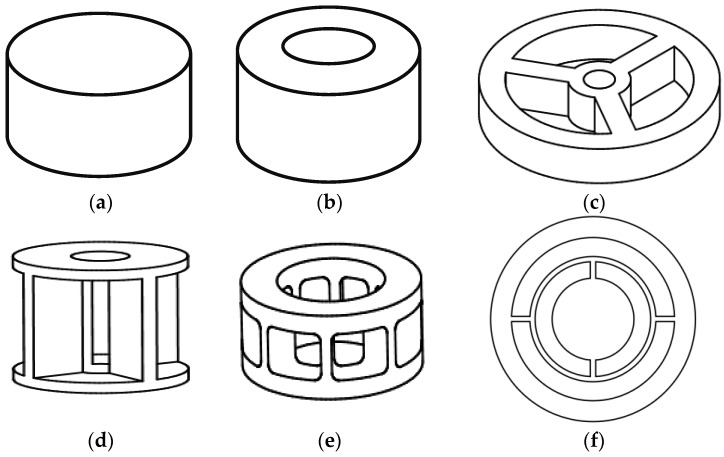
Various structures of torque sensor. (**a**) solid cylinder; (**b**) hollow cylinder; (**c**) hub sprocket; (**d**) hollow cruciform; (**e**) hollow hexaform; (**f**) spoke topology.

There are many previous studies on joint torque sensors. Figure 3 shows various types of these torque sensors. Solid [9] (Figure 3a) and hollow [10] (Figure 3b) cylinders are simple and rigid structures. Designs researched to increase the sensor sensitivity include the hub-sprocket design [11] (Figure 3c), the hollow cruciform design [12] (Figure 3d) and the hollow hexaform sensor [13] (Figure 3e). Torque sensors with spoke topology [14] (Figure 3f) provide high sensitivity. However, the resulting compliance introduces a joint angle error that should be minimized. As a modification of the hub-sprocket design, the sensor of HIT (Harbin Institute of Technology) [15] was designed based on shear strain theory and the sensor of LWRIII [16] had four beams with a hole to increase sensitivity.

In previous research studies, the sensitivity and the stiffness of torque sensors have trade-off characteristics. To increase the sensitivity of the sensor, the stiffness has to be sacrificed. High torsional stiffness is important because the deflection by sensors adds position errors that cannot be compensated by the joint servo controller. However, it is desirable to design a sensor structure that generates a large strain for a given payload to increase the Signal-to-Noise (S/N) ratio and sensitivity of the sensor. Therefore there are two conflicting requirements: high stiffness and high sensitivity for torsion. In this paper, a new torque sensor with high sensitivity (TSHS) is proposed to resolve this problem.

This paper is organized as follows. In Section 2, the proposed torque sensor with high sensitivity is explained. Section 3 describes simulation modeling including finite element analysis (FEA) and optimization. Experimental validation of the sensor is presented in Section 4. Conclusions are drawn in Section 5.

## 2. Torque Sensor with High Sensitivity

**Figure 4:**
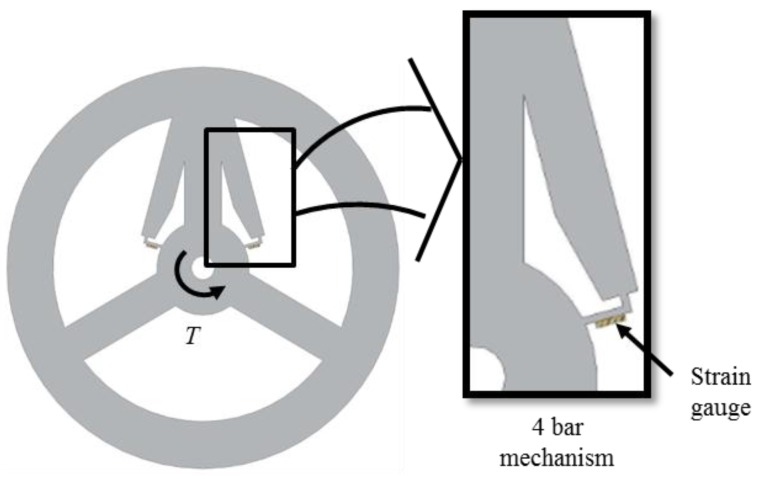
Torque sensor with high sensitivity (TSHS).

To increase sensitivity without sacrificing stiffness, a new torque sensor with high sensitivity (TSHS) is proposed as shown in Figure 4. The TSHS is a 3-beam torque sensor with a 4-bar linkage. The support portion of the TSHS is separated from the sensing portion. The stiffness is related to the 3-beam support portion while the sensitivity is related to the 4-bar linkage sensing portion. External torque causes the strain detected by the sensing portion of the sensor. The strain is measured by a strain gauge. The sensitivity can be increased, irrespective of the stiffness.

**Figure 5:**
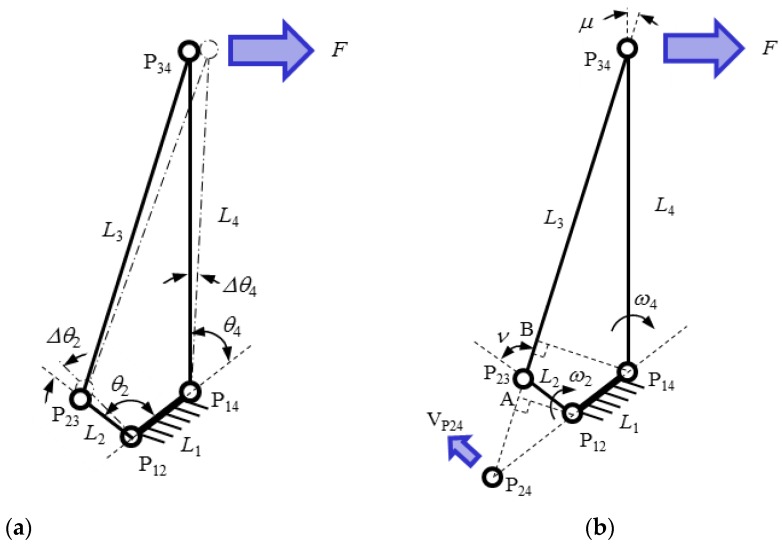
4-bar linkage mechanism. (**a**) small rotation of 4-bar linkage, (**b**) instant center of 4-bar linkage

The key idea for increasing the sensitivity comes from the 4-bar linkage mechanism as shown in Figure 5. In the Figure 5a, assume that the long link L_4_ rotates $\Delta \theta_{4}$ by the external force *F* at point P_34_ of the 4-bar linkage. The motion is transferred to the short link L_2_ by connecting link L_3_. If $\Delta \theta_{4}$ is small, the ratio of the angular displacement of L_2_ ($\Delta \theta_{2}$) and $\Delta \theta_{4}$ is the same as the angular velocity ratio as follows

(2) $$\frac{\Delta \theta_{2}}{\Delta \theta_{4}}=\frac{\omega_{2}}{\omega_{4}}$$

where, $\omega_{2}$ and $\omega_{4}$ are the angular velocities of each link L_2_ and L_4_, respectively.

The angular velocity ratio theorem can be used to obtain the angular velocity ratio [17]. In the Figure 5b, point P_24_ is the instant center common to links 2 and 4. Its velocity V_P24_ is the same whether P_24_ is considered as a point of link 2 or of link 4. We can write

(3) $$V_{p_{24}}=\omega_{2}\cdot l_{P_{24}P_{12}}=\omega_{4}\cdot l_{P_{24}P_{14}}$$

where, *l*_p24p12_ is distance from P_12_ to P_24_ and *l*_p24p14_ is distance from P_14_ to P_24_. From Equation (3), the angular velocity ratio is as follows

(4) $$\frac{\omega_{2}}{\omega_{4}}=\frac{l_{P_{24}P_{14}}}{l_{P_{24}P_{12}}}$$

In the Figure 5b, triangle P_12_P_24_A and triangle P_14_P_24_B are similar triangles. The angular velocity ratio can be obtained as follows

(5) $$\frac{\Delta \theta_{2}}{\Delta \theta_{4}}=\frac{\omega_{2}}{\omega_{4}}=\frac{l_{P_{24}P_{14}}}{l_{P_{24}P_{12}}}=\frac{l_{4}\sin\mu }{l_{2}\sin\nu }$$

where l_2_ and l_4_ are the length of each link L_2_ and L_4_ respectively, and μ and ν are transmission angle between each link. If $l_{4}\sin\mu$ is larger than $l_{2}\sin\nu$, $\Delta \theta_{2}$ is larger than $\Delta \theta_{4}$.

The 4-bar linkage shape of TSHS is different from the ideal 4-bar linkage mechanism. There are no real joints. If the links are too strong, amplification by the 4-bar linkage shape does not work. Hence, L_2_ is thinly designed. Links receive a bending moment as well as compression/tension forces. Bending by $\Delta \theta_{2}$ and $\Delta \theta_{4}$ produce a strain force on each link of the sensor. When $\Delta \theta_{2}$ is larger than $\Delta \theta_{4}$, strain of L_2_ is larger than that of L_4_.

When the external moment force is loaded on the torque sensor, the sensitivity of the torque sensor is proportional to the strain on the strain gauge attached to short link L_2_ of the 4-bar linkage shape while the stiffness of the torque sensor is proportional to the angular deformation of the sensor. The sensitivity and stiffness of the torque sensor are defined as follows

(6) $$K_{sensitivity}=\frac{\varepsilon }{n_{z}}$$

(7) $$K_{stiffness}=\frac{n_{z}}{\delta }$$

where ε is the strain of the strain gauge attached to short link L_2_ of the 4-bar linkage shape, δ is the angular deformation of the sensor and n_z_ is the external moment. The performance index λ is defined as the product of the sensitivity and the stiffness as follows

(8) $$\lambda =K_{sensitivity}•K_{stiffness}=\frac{\varepsilon }{n_{z}}•\frac{n_{z}}{\delta }=\frac{\varepsilon }{\delta }$$

## 3. Simulation

### 3.1. Finite Element Analysis Model

The joint torque sensors are usually assembled with reducers. The stiffness of the joint is related with not only the torque sensor but also the reduction gear and the link. The characteristics of the entire joint is another research topic. In this paper, the stiffness of the torque sensor is discussed. Torque sensors are matched with the reduction gear with rated torque of 40 Nm (harmonic driver SHG-32-80-2UH) [18].

**Figure 6:**
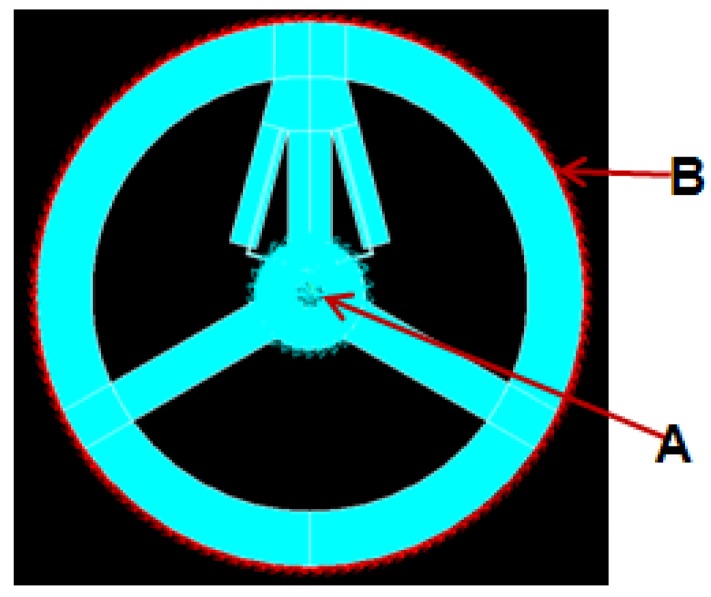
Boundary condition of the TSHS.

The boundary condition of the TSHS is defined as shown in Figure 6. The inner part is fixed and the external torque 40 Nm is loaded on outer surface B.

**Figure 7:**
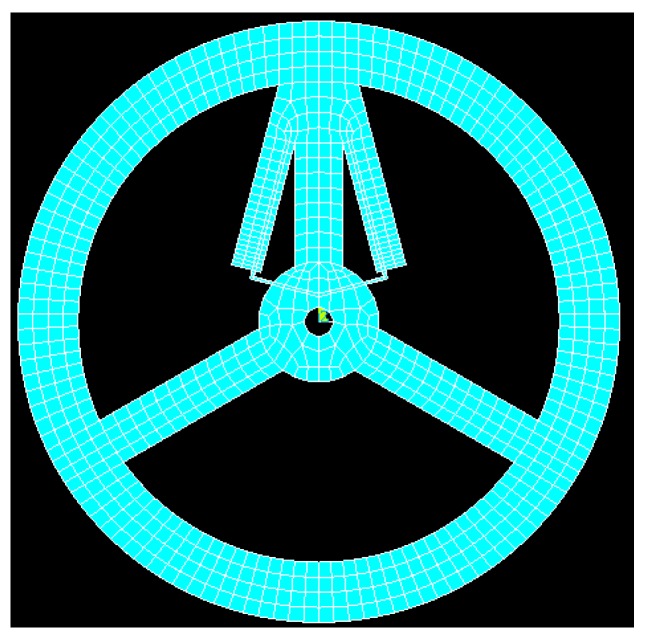
Mapped Mesh Model of the TSHS.

The material comprising the sensor is hard aluminum alloy AL6061-T6. The material parameters are as follows: the elastic modulus E=68,900 Pa, Poisson’s ratio μ=0.33 and the density ρ=2.71 g/cm^3^. In this paper, FEA software ANSYS [19] is used to analyze the torque sensor. Figure 7 shows mapped mesh of TSHS using the element solid 45. There are 2712 elements and 4480 nodes.

### 3.2. The Optimization

**Figure 8:**
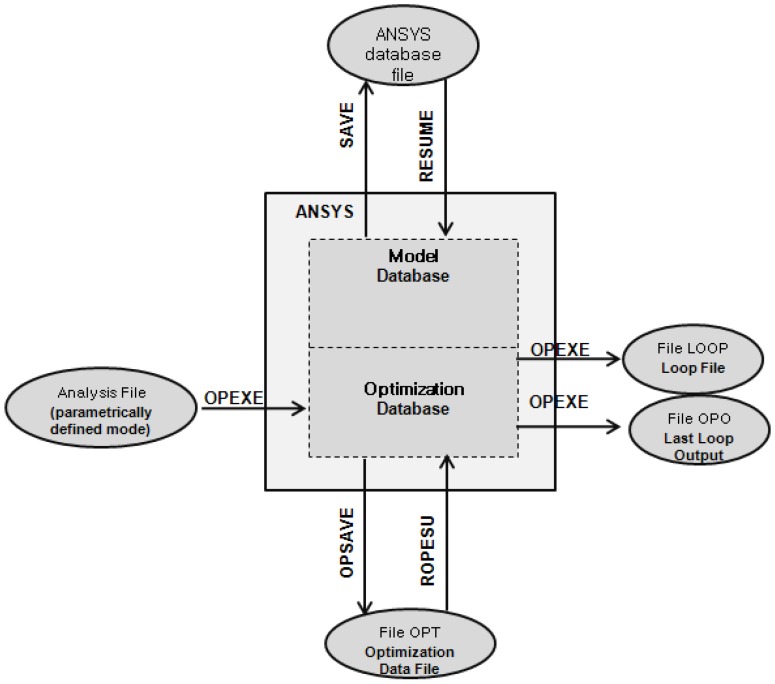
Optimization data flow.

The TSHS has many design variables and the sensitivity of the TSHS is related to these variables. The optimization by the ANSYS software is used to maximize the sensitivity of the TSHS. The ANSYS optimization routines use three types of variables that characterize the design process: design variables (DV), state variables (SV) and objective variables (OV). These variables are represented by scalar parameters in the ANSYS Parametric Design Language (APDL) [19]. Figure 8 shows the optimization data flow.

**Figure 9:**
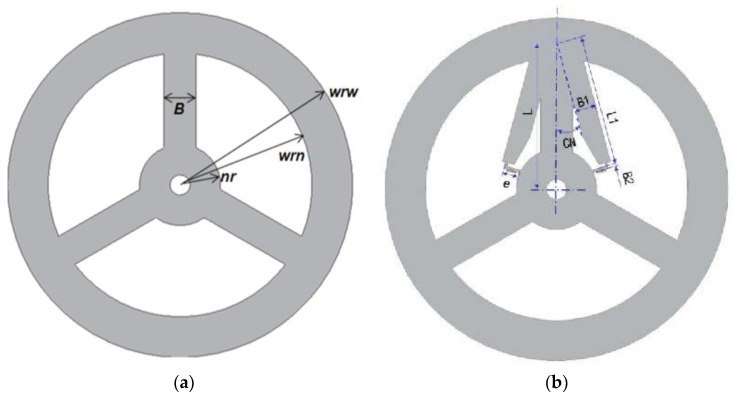
Design variables of torque sensors. (**a**) 3-beam torque sensor; (**b**) TSHS.

In this paper, a 3-beam torque sensor and the TSHS are compared. Fixed parameters and design variables (DV) of the two models are shown in Figure 9 and Table 1. Fixed parameters (wrw, wrn, nr, B) are the same for the two sensors. The link length (e) where the strain gauge is attached has to be longer than the strain gauge length. In this research, it is determined to be 4.5 mm. Design variables (L, B1, L1, B2, CN) have initial values and upper and lower limits, described in Table 1.

To improve the sensitivity without reduction of the stiffness of the torque sensor, the strain ε and the deformation δ of the torque sensor are used as SV while the strain ε is selected as OV.

### 3.3. FEA Results

**Figure 10:**
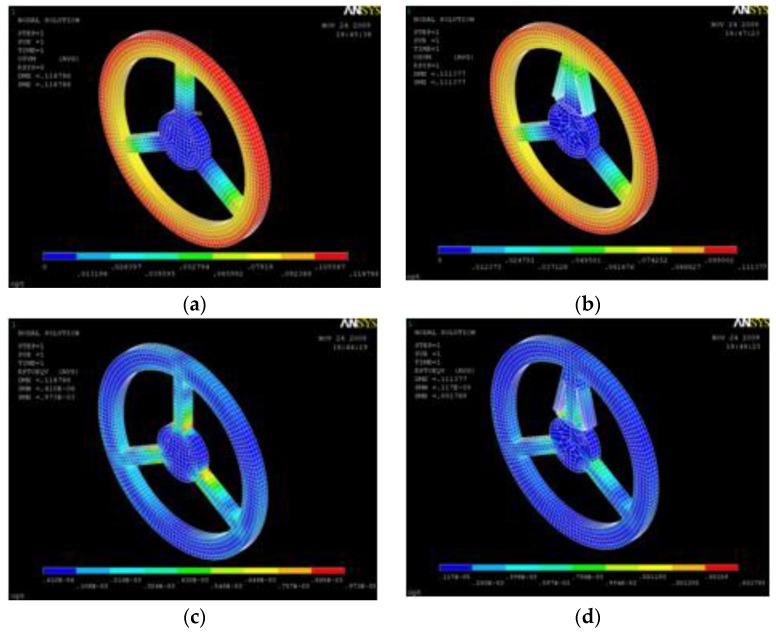
Deflection and strain FEA results of torque sensors. (**a**) Deflection of 3-beam sensor; (**b**) Deflection of TSHS; (**c**) strain of 3-beam sensor; (**d**) strain of TSHS.

After optimization to improve the sensitivity of the torque sensor, the optimal design of both the TSHS and FEA results are shown in Table 2. Figure 10 shows deflection and strain FEA results of the torque sensors. Deformation results are similar while strain of the TSHS is larger than that of 3-beam sensor.

## 4. Experimental Results

### 4.1. Experimental Equipment

**Figure 11:**
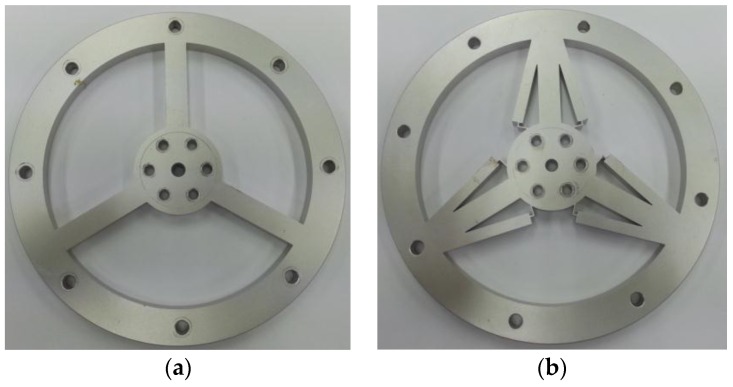
Torque sensors. (**a**) 3-beam torque sensor; (**b**) TSHS.

The actual 3-beam torque sensor and TSHS are both constructed to verify validity of the proposed mechanism as shown in Figure 11. Both actual torque sensors include holes for bolting. A pair of 4-bar linkage shape sensing portions is attached to each supporting beam of the TSHS for symmetry. Strain gauges are attached to sensing portions of the torque sensor. Table 3 shows specifications of the strain gauge. The Strain gauge was measured by NI 9237 of National Instruments.

**Figure 12:**
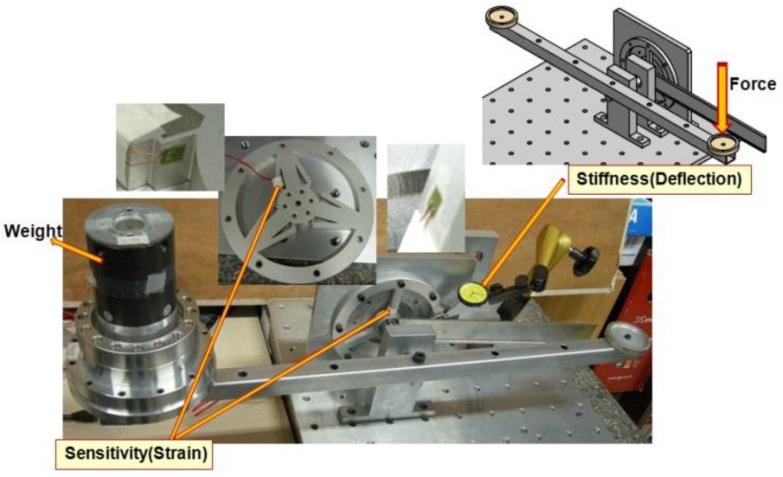
Experimental equipment.

**Figure 13:**
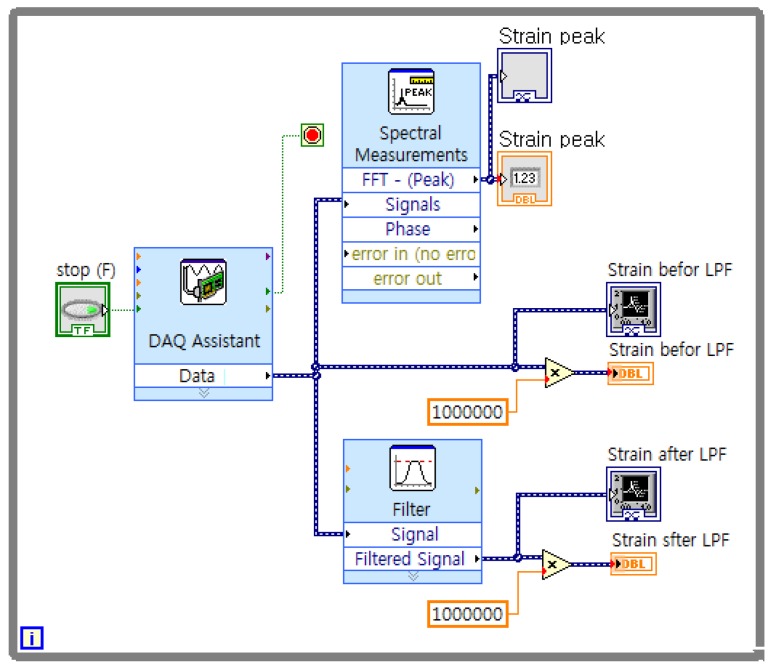
Program in LabVIEW.

Figure 12 shows the experimental equipment. The experimental equipment consists of the torque sensor, horizontal bar, mass, dial gauge and strain gauge measurement system. Data is captured by the program LabVIEW [20] as shown Figure 13.

### 4.2. Experimental Results

To verify the FEA result of Section 3, the two sensor models were tested for strain and deflection. Table 4 shows the FEA results of Section 3 and experimental results of the two models, the 3-beam sensor and the TSHS. As shown in the Table 4, the experiment results are similar with the FEA results. The sensitivity (strain) of the TSHS is 3.5 times higher than the sensitivity of the 3-beam torque sensor. The deflection of the TSHS is reduced by 7%. That means that the stiffness of the TSHS also increases. The performance index λ of TSHS has been improved by a factor of 3.8.

**Figure 14:**
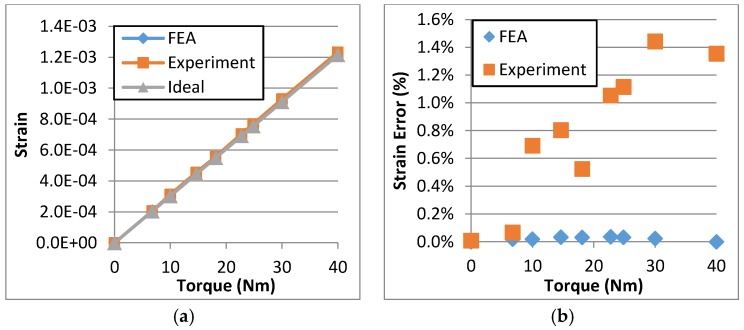
Relationship of the strain vs torque. (**a**) strain; (**b**) strain error.

By analyzing the data of the FEA and the experimental results, the ideal strain, the FEA strain and the experimental strain with respect to the torque input can be obtained as shown in Figure 14. Figure 14a shows that the FEA results and the experimental results are similar with the ideal results. In Figure 14b, the linearity error is below 1.5%. In this range, it illustrates that the TSHS exactly reflects the payload.

## 5. Conclusions

In this paper, in order to improve the sensitivity of torque sensors without compromising stiffness, a new concept of torque sensor with high sensitivity (TSHS) is proposed. The key idea of the TSHS comes from the 4-bar linkage in which the angular displacement of the short link is enlarged. Because the part of the sensor related to the sensitivity is separated from the part related to the stiffness of the sensor, the sensitivity can be improved without decreasing stiffness.

The proposed mechanism is analyzed by FEA using ANSYS. To maximize the performance of the sensor, the proposed mechanism has been optimized. The actual TSHS is constructed to verify validity of the proposed mechanism.

The sensitivity (strain) of the TSHS is 3.5 times higher than the sensitivity of the 3-beam torque sensor. The deflection of the TSHS is also reduced by 7%. That means that the stiffness of the TSHS also increased. The performance index λ of TSHS has been improved by a factor of 3.8. The TSHS has a linearity error below 1.5%.

As a further study, this concept of the TSHS can be extended to research multiple DOF torque/force sensors.

## Figures and Tables

**Table 1:** Parameters of the TSHS.

		3 Beams	TSHS		
			Initial Value	Lower Limit	Upper Limit
Fixed Parameter	wrw (mm)	75			
	wrn (mm)	60			
	nr (mm)	20			
	B (mm)	10			
	e (mm)	-	4.5		
DV	L (mm)	-	64	63	70
	B1 (mm)	-	5.0	3.8	6.0
	L1 (mm)	-	47.5	45	50
	B2 (mm)	-	0.5	0.4	0.6
	CN (deg)	-	15	14	15.5

**Table 2:** The optimal design and FEA results of TSHS.

		3 Beams	TSHS	
			Initial Value	Optimal Result
DV	L (mm)	-	64	65
	B1 (mm)	-	5.0	5.0
	L1 (mm)	-	47.5	47.5
	B2 (mm)	-	0.5	0.55
	CN (deg)	-	15	15
SV	ε	3.51 × 10^−4^	6.70 × 10^−4^	12.2 × 10^−4^
	δ (rad)	21.6 × 10^−4^	21.6 × 10^−4^	19.8 × 10^−4^
OV	ε	3.51 × 10^−4^	6.70 × 10^−4^	12.2 × 10^−4^
	λ	0.163	0.310	0.615

**Table 3:** Specification of the strain gauge.

Parameters	Contents
Strain gauge type	KFG-02-120-C123L1M2R
Gauge Factor	2.25 ± 1.0%
Gauge Resistance	119.6 ± 0.4 Ω
Gauge size	3.3 × 2.4 mm

**Table 4:** Comparison experiment result with FEA.

Name	Method	Strain	Deflection (rad)	*λ*
3 beams	FEM	3.51 × 10^−4^	21.6 × 10^−4^	0.163
	Experiment	3.48 × 10^−4^	22.0 × 10^−4^	0.158
	Error	−0.85%	1.85%	−2.66%
TSHS	FEM	12.2 × 10^−4^	19.8 × 10^−4^	0.615
	Experiment	12.3 × 10^−4^	20.5 × 10^−4^	0.601
	Error	1.40%	3.80%	−2.31%
$\frac{\text{TSHS}}{3\mathrm{beams}}$	FEM	3.48	0.916	3.77
	Experiment	3.53	0.932	3.80
